# 2018 in review: five hot topics in tropical medicine

**DOI:** 10.1186/s40794-019-0082-z

**Published:** 2019-04-15

**Authors:** Leila Makhani, Aisha Khatib, Antoine Corbeil, Ruwandi Kariyawasam, Hira Raheel, Shareese Clarke, Priyanka Challa, Emma Hagopian, Sumontra Chakrabarti, Kevin L. Schwartz, Andrea K. Boggild

**Affiliations:** 1 Department of Family and Community Medicine, University of Toronto, Toronto, Canada; 2 Tropical Disease Unit, Toronto General Hospital, 200 Elizabeth Street, 13EN-218, Toronto, ON M5G 2C4 Canada; 3 Department of Laboratory Medicine and Pathobiology, University of Toronto, Toronto, Canada; 4 Institute of Medical Science, University of Toronto, Toronto, Canada; 5 Faculty of Medicine, University of Toronto, Toronto, Canada; 6 Department of Life Science, University of Toronto, Toronto, Canada; 7 Department of Arts and Science, University of Toronto, Toronto, Canada; 8 Department of Medicine, University of Toronto, Toronto, Canada; 9 Division of Infectious Diseases, Trillium Health Partners, Mississauga, Canada; 10 Division of Infectious Diseases, St. Joseph’s Health Centre, Toronto, Canada; 11 Dalla Lana School of Public Health, University of Toronto, Toronto, Canada; 12 Public Health Ontario, Toronto, Canada

**Keywords:** Human African trypanosomiasis, Ivermectin, Monkeypox, *Plasmodium vivax*, Strongyloidiasis, Tafenoquine, Yellow fever

## Abstract

The year 2018 heralded many new developments in the field of tropical medicine, including licensure of novel drugs for novel indications, licensure of existing drugs for existing indications but in novel settings, and globalized outbreaks of both vector-borne and zoonotic diseases. We herein describe five top stories in tropical medicine that occurred during 2018, and illuminate the practice-changing development within each story.

## 2018 in review: five hot topics in tropical medicine

As we move further into the year 2019, reflection on the successes and challenges of 2018 enables better understanding of the complex landscape of tropical medicine, prioritize initiatives that might dovetail with last year’s successes and capitalize on their momentum, and, finally, identify areas for collective renewed energy and focus. In this review, we will illuminate five hot topics in tropical medicine that made an impact in our field last year. For each hot topic, we aim to situate the development within the travel and tropical medicine literature, note the scale of impact, identify what changed in 2018, and interpret how our practice and approach will be altered going forward.

## Hot topic 1: approval of ivermectin for strongyloidiasis by Health Canada

### Scope of impact: regional, North America

#### Background

In 2015, the Nobel Prize in Physiology or Medicine was jointly awarded to Drs. William C. Campbell and Satoshi Ōmura for their discovery of the avermectin family of compounds, from which ivermectin (22, 23-dihydroavermectin B1) is derived [1]. As a macrocyclic lactone, ivermectin primarily acts by potentiating the glutamate-gated chloride channels of neural and muscle cells present in invertebrates, interfering with their polarization and ultimately leading to paralysis [2]. In humans, ivermectin is well tolerated, with the most frequent adverse reaction being pruritus, although caution is indicated due to the medication’s potentiation of GABAergic synapses which occasionally leads to other neurotoxicity reactions such as headaches, seizures, and central nervous system depression [3]. Crossing of the blood-brain barrier occurs with higher doses of the medication [4]. Specific contraindications for ivermectin include a high microfilarial burden in patients with loiasis, due to its association with severe adverse events such as fatal encephalopathy [5], and pregnancy due to lack of safety studies in humans with potential teratogenic effect in animals (Category C as per the Food and Drug Administration) [6]. Ivermectin is presently licensed for human use in several countries for a variety of parasitic indications, particularly onchocerciasis, due to *Onchocerca volvulus*, the blackfly-vectored cause of River Blindness, and strongyloidiasis, due to the threadworm *Strongyloides stercoralis* (Table 1).

**Table 1:** First market approvals and current indications of ivermectin by country

Region	First Approval Date	Current Indications
France^a^	October 1987	Onchocerciasis, strongyloidiasis, lymphatic filariasis, human sarcoptic scabies
United States^b^	November 1996	Onchocerciasis, strongyloidiasis
Australia^c^	June 1997	Onchocerciasis, strongyloidiasis, human sarcoptic scabies, crusted scabies
Japan^d^	October 2002	Strongyloidiasis, human sarcoptic scabies, crusted scabies
Netherlands^e^	March 2003	Strongyloidiasis, lymphatic filariasis, human sarcoptic scabies
New Zealand^f^	August 2005	Strongyloidiasis, lymphatic filariasis, human sarcoptic scabies
Germany^a^	February 2016	Strongyloidiasis, lymphatic filariasis, human sarcoptic scabies
Canada^g^	September 2018	Onchocerciasis, strongyloidiasis

^a^European Medicines Agency. Ivermectin (systemic use): List of nationally authorized medicinal products. Human Medicines Evaluation Division. Available at (accessed February 3, 2019): [https://www.ema.europa.eu/documents/psusa/ivermectin-systemic-use-list-nationally-authorised-medicinal-products-psusa/00002959/201502_en.pdf]

^b^Food and Drug Administration. Labelling and Clinical Review of Final Printed Labelling: Stromectol (Ivermectin) 3 mg tablets (New Formulation). Center for Drug Evaluation and Research. 2000. Available at (accessed February 3, 2019): [https://www.accessdata.fda.gov/drugsatfda_docs/nda/98/50-742s001_Stromectol_AdminCorres.PDF]

^c^Therapeutic Goods Administration. AusPAR Product Information: Stromectol Tablets. 2013 Jul; Accessible at (accessed February 3, 2019): [https://www.tga.gov.au/sites/default/files/auspar-ivermectin-131030-pi.pdf]

^d^Pharmaceuticals and Medical Devices Agency. FY2006 List of Approved Products: New Drugs. 2006; Available at (accessed February 3, 2019): [https://www.pmda.go.jp/files/000153730.pdf]

^e^Medicines Evaluation Board. Public Assessment Report – Scientific Discussion: Ivermectin Substipharm 3 mg tablets. 2018 Jul; Accessible at (accessed February 3, 2019): [https://db.cbg-meb.nl/Pars/h120488.pdf]

^f^Medsafe. Medsafe Product Detail: Stromectol tablet 3 mg. 2018 Feb; Accessible at (accessed February 3, 2019): [https://www.medsafe.govt.nz/regulatory/ProductDetail.asp?ID=11299]

^g^Health Canada. Drug and health product submissions under review (SUR). Government of Canada. 2018. Available at (accessed February 3, 2019): [https://www.canada.ca/en/health-canada/services/drug-health-product-review-approval/submissions-under-review.html]

##### Ivermectin in the Canadian context

In Canada, the incidence of imported onchocerciasis is vanishingly small, with only 2 cases recorded over a 6-year period at a large referral centre servicing a diverse catchment population [7]. Although imported onchocerciasis may be less rare in other non-endemic countries [8], a more common condition requiring ivermectin treatment in Canada is strongyloidiasis, a disease particularly seen among migrants and refugees, who account for 21.9% of the total Canadian population. Of these 7.5 million foreign-born Canadians representing the country’s ethnocultural diversity, 85% emigrated from regions endemic for strongyloidiasis [9]. Amongst our migrant population, studies show a wide range of seroprevalence rates for strongyloidiasis, with up to 77% of refugees from Cambodia and 12% of refugees from Vietnam demonstrating serologic reactivity [10, 11]. Additionally, strongyloidiasis is consistently among the top five causes of illness in surveillance studies of new migrants presenting for care at specialized post-travel centres [12, 13]. Assuming an average source-country prevalence rate of 40%, at least 2.5 million new Canadians suffer from simple intestinal strongyloidiasis [10]. In addition to strongyloidiasis, hookworm-related cutaneous larva migrans is repeatedly demonstrated to be a top cause of imported dermatologic morbidity in Canadians, and is easily treated with a single dose of ivermectin [14, 15].

#### Landscape of Ivermectin in Canada prior to 2018

There is an unequivocal lack of access to essential antihelminthic drugs in Canada. Effective medications are neither licensed nor marketed in the country for the vast majority of neglected tropical diseases (NTDs) listed by the World Health Organization (WHO) [16]. To obtain some of these indispensable treatment options, including ivermectin prior to 2018, clinicians are required to apply to Health Canada’s Special Access Programme (SAP), which provides individual approvals for unlicensed medicinal products in the country. However, accessibility remains limited, with time from submission of the SAP application to receipt of the medication often surpassing a week [17]. Such delays to accessing potentially life-saving drugs led to high-profile reports in news media of deaths related to disseminated strongyloidiasis that were attributed to inappropriate access to ivermectin [18].

##### Implications of strongyloidiasis

Although occasionally presenting as an acute infection in short-term travellers [19], overt manifestations of strongyloidiasis are often absent, subtle, or very non-specific, becoming apparent only decades after exposure. Chronic presentations can include isolated eosinophilia, refractory gastritis, abdominal pain, or chronic pruritic migratory skin lesions (larva currens) [20–22]. The most severe forms of strongyloidiasis are paradoxically attributable to its insidious and autoinfective nature, since undiagnosed, lifelong infections can result in a fatal dissemination syndrome when the Th2 immune response becomes impaired by immunosuppressive medications or viral co-infections, such as HTLV-1 [10, 23]. The trifecta of increasing migration from high-prevalence source countries, ever-expanding use of iatrogenic immunosuppression in the care of Canadian patients with many different types of inflammatory and malignant illnesses, and the lack of access to ivermectin culminated in frequent cases of fatal disseminated strongyloidiasis across Canada. These occurrences motivated the Committee to Advise on Tropical Medicine and Travel (CATMAT), an external advisory body to the Public Health Agency of Canada, to draft national guidelines on the subject in order to reduce country-wide morbidity and mortality [10].

#### What changed in 2018?

After submission for approval by Merck & Co., Inc., ivermectin (as Stromectol®) was granted a Notice of Compliance by Health Canada, the country’s regulatory body for licensure of pharmacologic products, on September 10, 2018, which enables the drug to be marketed and maintained on pharmacy formularies [24]. Health Canada granted approval for use of ivermectin for the indications of strongyloidiasis and onchocerciasis, meaning that the use of ivermectin remains off-label for other conditions treatable with ivermectin, including human sarcoptic scabies, crusted scabies, lymphatic filariasis, and cutaneous larva migrans. Undoubtedly, this approval will facilitate more timely access to ivermectin for treatment of both simple and severe strongyloidiasis, which is likely to reduce related morbidity and mortality, particularly as it might be better integrated into pre-immunosuppression screening programs (e.g., in advance of solid organ transplant or cancer chemotherapy). Similarly, off-label treatment can now be expedited for cutaneous larva migrans in primary care settings, where most such patients are encountered [25]. Ivermectin accessibility in the context of scabies outbreaks at long-term care facilities could also potentially provide infection prevention and control benefits unrealized prior to 2018 due to the lack of drug availability. A potential drawback of ivermectin approval in Canada remains the financial constraints facing patients who lack public or private drug coverage, for whom the medication will now cost $7 Canadian per pill, whereas via the federal SAP, the drug was dispensed free of charge.

#### Conclusions

The antiparasitic medication ivermectin has had a major impact on the burden of NTDs such as onchocerciasis on the global scale. In Canada, ivermectin’s relative inaccessibility has left a substantial at-risk population without a curative option for strongyloidiasis, a disease estimated to impact up 2.5 million foreign-born Canadians nationwide. With the approval for licensure and marketing of ivermectin in September 2018, clinicians caring for individuals with many parasitic infections, including strongyloidiasis, are now able to appropriately treat them. While financial barriers may exist for a proportion of Canadian patients without federal, provincial, or private drug coverage, this represents a promising milestone for Canadians, and a potential example for other countries to likewise expand accessibility to essential antihelminthic medications.

## Hot topic 2: yellow fever exported by international travelers

### Scope of impact: global

#### Background

Yellow Fever (YF) is an arbovirus from the genus Flavivirus, with non-human and human primate reservoirs, transmitted by infected *Aedes* and *Haemogogus* mosquitoes [26]. YF is endemic in 29 sub-Saharan African countries, and in 13 countries of tropical South America, resulting in an estimated 200,000 cases with 30,000 annual deaths in endemic populations [26]. Three transmission cycles exist: sylvatic, with primate-to-human transmission; intermediate, with primate- or human-to-human transmission; and lastly urban, consisting of human- and peri-domestic mosquito transmission [26–28]. Transmission often occurs when humans encroach jungle borders for occupational or recreational activities [26–28].

##### Clinical presentation

It is estimated that YF causes mild disease in 80–90% of infections situated in endemic or transmission areas, with severe disease manifesting in ~ 15% [28–30]. Poor diagnostics in endemic areas, multiple competing co-endemic flaviviruses affecting performance of serologic testing, and variable immunization scheduled in risk areas hinder our understanding of the true seroprevalence of infection. Symptomatic infection lasts 3–6 days with sudden onset of fever, chills, headache, myalgia, prostration, nausea, and vomiting. After a period of remission of hours to days, the second phase of disease may present with fever, jaundice, hemorrhagic symptoms, shock and multisystem organ failure [28]. Case fatality for individuals with hepatorenal dysfunction is 20–50% [28–30]. Presumptive diagnosis of YF is often based on clinical features (including febrile jaundice), travel history, activities engendering mosquito exposure, and epidemiological history [31]. Given the wide differential diagnosis that includes other severe tropical infections such as malaria and leptospirosis, laboratory confirmation is required [31]. While treatment of YF has historically consisted of supportive and critical care management, small scale data are emerging out of Brazil suggesting that sofosbuvir, a drug used to treat hepatitis C, another flavivirus, might have clinical efficacy [32].

#### Landscape of YF in travelers prior to 2018

##### Prevention in travelers

Prevention of YF is achieved through mosquito avoidance, breaking perpetuating transmission cycles, and most effectively, vaccination [33]. Vaccination is recommended for all eligible persons aged ≥9 months, traveling to places where YF transmission occurs, and given at least 10 days before travel [33–37]. Since 2016, the WHO has advised that a single dose of YF vaccine confers life-long protection and that boosters are unnecessary, except in some immunocompromised populations [38, 39]. For most travelers, one dose of YF vaccine provides long-lasting protection [33, 35–38, 40]. Individuals traveling to areas with YF outbreaks may consider a booster dose if over 10 years have elapsed since last vaccination [33, 35, 40]. Travelers who have never been vaccinated against YF should avoid traveling to endemic or outbreak areas [33, 35, 40, 41].

##### Risk for travelers

The estimated YF risk in travelers is low, ranging between < 1 per 1,000,000 to < 1 per 100,000 travelers per month of travel [34]. The rate of disease per number of travelers for a 2-week stay during a high-risk season is estimated at 50 per 100,000 and 5 per 100, 000 travelers in Africa and South America, respectively [34]. Risk is often determined by vaccination status, location of travel, season, duration of exposure, occupational and recreational activities, as well as the local rate of transmission [34]. In unimmunized travelers, the case-fatality rate is high, approaching up to 90% [42].

#### What changed in 2018?

In the year 2018, the potential for brand new urban cycles of transmission, an increase in the number of exported cases from risk areas by travelers, some of whom had fatal outcomes, and the ongoing threat to YF vaccine supply chain in the context of high demand, all culminated in the development of new public health-level policies and guidelines in non-endemic areas such as Canada and the US.

##### Concern for new urban cycles of transmission

Recently, YF outbreaks have increased, resulting in cases being exported by travelers to non-endemic countries. In 2016, 11 long-term travelers from Asia acquired YF after visiting Angola, where one of the largest urban outbreaks was occurring [43]. Travelers were unvaccinated or had received late vaccination, and returned to Asia while infected [43]. With the presence of the urban mosquito vector *Aedes aegypti* among large unvaccinated populations, this exportation of YF to Asia via international travelers put 1.8 billion individuals at risk, by introducing a possible new urban cycle of transmission [43]. Between 1 July 2017 and 24 April 2018, a total of 1218 confirmed human cases of YF, including 364 deaths, were observed in Brazil [27, 44]. This outbreak occurred in densely populated metropolitan areas, including Rio de Janeiro and Sao Paulo, encompassing a population of over 32 million inhabitants [27, 44]. Until April 2017, these areas were deemed of no risk for YF virus transmission [27, 44]. As of January 2018, the WHO updated YF vaccination recommendations for Brazil to include all persons traveling to or living in: all of Espirito Santo State, São Paulo State, Rio de Janeiro State, Paraná State, Santa Catarina State, and Rio Grande do Sul State [44]. Since the vaccination campaign was employed, only 53.6% of São Paulo, 55.6% of Rio de Janeiro, and 55% of Bahia states achieved acceptable levels of vaccination [44], supporting the potential for ongoing YF transmission.

##### Exported travel-related cases

Between January and March 2018, 10 travel-related cases of YF, including four deaths, were reported in international travelers returning from Brazil, all of whom were unvaccinated [42]. Five of the cases involving travelers from Argentina and Chile were reported by the Program for Monitoring Emerging Diseases (ProMED) [42]. The other five confirmed cases were reported by GeoSentinel, the global clinician-based sentinel surveillance system for travel-related illness among international travelers and migrants, a first for the surveillance system [42]. All of the cases were acquired via sylvatic transmission and travel time varied between 1 and 4 weeks [42]. As of 24 April 2018, a total of 19 confirmed cases of YF were reported among unvaccinated international travelers in Brazil, Argentina, France, Germany, the Netherlands, Romania, Switzerland, and the United Kingdom [45]. In comparison, from 1970 through 2015, only 10 cases of YF were reported in unvaccinated travelers from the United States and Europe to West Africa and South America [34].

##### Novel approach to disruption in vaccine supply

The YF vaccine YF-Vax, produced by Sanofi Pasteur, is an effective, live-attenuated vaccine licensed and marketed in North America [33, 35, 36]. As of November 2015, the confluence of greater demands stemming from the Angola outbreak, and the transition of YF-Vax production to a newer Sanofi Pasteur facility, led to critical vaccine shortages that were felt around the world [35]. During this time, the US FDA negotiated access to and licensure of a similar vaccine, Stamaril, manufactured in Sanofi Pasteur France since 1986 and distributed in 70 countries with comparable safety and efficacy [35]. In Canada, Sanofi Pasteur did not distribute Stamaril, but provided a steady yet reduced supply of YF-Vax. As such, in 2018, CATMAT drafted guidelines recommending the use of fractional dosing as an interim mitigation strategy that would confer immunity for at least 12 months, despite being unable to fulfill IHR requirements for an international certificate of vaccination [35, 46]. Fractional dosing of one-fifth the dose (0.1 ml SC), of YF-Vax is recommended as an alternative personal protection measure, with the issuance of a waiver indicating vaccine shortage [35].

#### Conclusion

The ongoing YF-Vax shortage will continue to challenge our collective ability to prevent new cases both locally and amongst travelers, break known transmission cycles in endemic areas, and issue vaccine certificates that are fully compliant with IHRs. In addition to strengthening vaccine coverage in YF endemic countries, the emphasis remains on ensuring vaccination of individuals crossing borders to prevent international spread and to protect travelers from disease [37, 43]. Each year millions of travelers depart from YF endemic areas to non-endemic areas that have potential for virus transmission without having to provide proof of vaccination [37, 43, 47]. Rapid global changes in human mobility and urbanization necessitate re-examination of vaccination policies and practices to prevent urban YF epidemics [37, 43]. Circumventing IHRs through fabrication of YF certificates without administration of vaccine is a matter of urgency requiring improved legislations and protocols [43]. Both enhanced laboratory capacity and augmented provider-based surveillance will better position the international community to respond promptly to new outbreak foci and to generate new knowledge around clinical aspects of disease, such as true incidence and prevalence, that remain elusive. Clinicians assessing ill returned travelers should remain vigilant for the possibility of YF in areas with ongoing transmission and outbreaks.

## Hot topic 3: fexinidazole for human African trypanosomiasis (HAT)

### Scope of impact: predominantly regional, west and sub-Saharan Africa

#### Background

Human African Trypanosomiasis (HAT), also known as sleeping sickness, is a fatal neglected tropical disease that affects approximately 70 million people living in sub-Saharan Africa. Although it can affect travelers returning from endemic areas, the large majority of people who develop the disease live in rural and isolated areas where health systems are often weak or non-existent [48–51]. Untreated disease leads to coma and eventual death [52]. HAT can affect both humans and animals, thereby causing major challenges in the areas of food security, health, agriculture and economic growth, and importantly, eradicability [48]. Considered a significant public health problem, the WHO has targeted elimination of HAT by 2020 [53].

Transmitted by tsetse flies, HAT is caused by protozoan parasites belonging to the *Trypanosoma* genus [48, 49, 54]. The two causative species are *Trypansoma brucei gambiense* and *T. b. rhodesiense*, with the former prevalent in west and central Africa, causing chronic infection over months to years, and accounting for approximately 97% of known cases. Conversely, *T. b. rhodesiense* is endemic to south and east Africa, leads to acute infection and represents no more than 3% of cases [48, 52]. Diagnosis and management of HAT is challenging, and some of the treatment regimens can be highly toxic or even fatal. In 2018, Mesu and colleagues published a promising trial in the Lancet demonstrating the safety and efficacy of oral fexinidazole for the treatment of late stage *T. b. gambiense* [55], which could alter the course of this fatal disease.

##### Clinical features of HAT

HAT has two distinct clinical stages known as the hemolymphatic (peripheral) and the meningoencephalic (central nervous system) stages [52, 55]. After an inoculation bite by a tsetse fly, the parasite enters the local lymphatics, and then, once in the bloodstream, the hemolymphatic stage begins. This stage is characterized by non-specific symptoms including malaise, undulating fever, headache, pruritus, and lymphadenopathy [52, 55]. Due to the chronic, indolent course of *T. b. gambiense*, patients can remain undetected in the asymptomatic stage for months to years, challenging case detection, while others will develop the classic posterior cervical lymphadenopathy (Winterbottom’s sign) enabling earlier identification [52]. Without timely diagnosis and treatment, the infection will progress to involve the central nervous system, advancing disease into the meningoencephalitic stage. Patients in this second stage will manifest several neurological symptoms such as headaches, confusion, severe sleep disturbances, personality changes, manic episodes, and eventually, coma and death [52, 55].

##### Diagnosis of HAT

Accurate diagnosis of HAT is limited by poor and labour intensive diagnostics, yet treatment is stage-dependent, creating a severe challenge to timely and appropriate management. Definitive diagnosis involves a lumbar puncture for cerebrospinal fluid (CSF) analysis, so as to discriminate early- from late-stage disease and guide therapeutic decisions [56]. Currently, risk-based screening programs employ the card agglutination test for trypanosomiasis (CATT) in populations exposed to *T. b. gambiense*. Once peripheral disease is confirmed, patients undergo systematic staging by lumbar puncture. Due to high rates of relapse, patients are ideally followed up to 18–24 months post-treatment with repeat lumbar punctures to ensure disease and parasitologic cure [48, 55]. Newer molecular techniques as well as non-invasive testing are on the horizon, which will advance the diagnostic approach and eliminate the need for invasive diagnostic and prognostic procedures [48].

#### Landscape of HAT treatment prior to 2018

Traditionally, the treatment of HAT involved four drugs including pentamidine, suramin, melarsoprol, and eflornithine, with the more recent addition of nifurtimox-eflornithine combination therapy (NECT). Pentamidine and suramin are still used to treat early stage disease with *T. b. gambiense* and *T. b. rhodesiense*, respectively, and are administered parenterally [57]. The only treatment for late stage *T. b. rhodesiense*, which was also used for late stage *T. b. gambiense* for decades, is melarsoprol, an arsenical causing a post-treatment reactive encephalopathy, which itself has an associated mortality rate of 5.9% [55–57]. Melarsoprol requires parenteral administration, hospitalization, and close monitoring due to toxicity. In 1990, eflornithine was licensed for the treatment of *T. b. gambiense* and offers a safer option than melarsoprol but still has a high adverse event profile. Like melarsoprol, eflornithine requires multiple daily infusions, often difficult in a remote or rural setting where HAT is endemic [56–58]. In 2009, NECT was introduced for *T. b. gambiense*, which involves a combination of oral and IV therapy and a simpler and more cost-effective regimen [58]. While NECT significantly decreased the number of incident cases, and is therefore deemed effective, it still requires hospital admission, sterile equipment for lumbar puncture, and trained health care professionals in extremely short supply in HAT endemic areas [56] (Table 2).

**Table 2:** HAT Treatment for Late Stage Disease from 1949 to 2018 [58]

Drug	Advantages	Disadvantages
Melarsoprol	Only treatment available for years, can be used for both g-HAT^a^ and r-HAT^b^	Derived from arsenic and can cause reactive encephalopathy, fatal in 3–10%
Eflornithine Monotherapy	Less toxic than melarsoprol	Can only be used for the treatment of g-HAT, requires 56 infusions over 14 days, inpatient admission, sterile equipment and trained hospital staff
Nifurtimox-Eflornithine Combination Therapy (NECT)^b^	Decreased the overall incidence of disease and relapse rates at 18 months as compared to eflornithine alone, and is less toxic than melarsoprol	Can only be used for the treatment of g-HAT (1st line), requires inpatient admission, sterile equipment and trained hospital staff

^a^ *g-HAT T. b. gambiense*

^b^ *r-HAT T. b. rhodesiense*

#### What changed in 2018?

In 2005, the antiparasitic drug called fexinidazole was rediscovered by the Drugs for Neglected Diseases initiative (DNDi) along with their partner affiliates, and is thought to be the first solely oral treatment effective for both early and late stages of *T. b. gambiense,* which led to investigation of its clinical utility in clinical trials [56, 59]. In January 2018, results of the multicenter, randomized, non-inferiority trial demonstrating safety and efficacy of oral fexinidazole for the treatment of late stage *T. b. gambiense* were published [55]. This trial enrolled 394 patients from *T. b. gambiense* treatment centres in the Democratic Republic of Congo (DRC) and the Central African Republic between 2012 and 2016. Patients were randomized to receive either fexinidazole or NECT therapy for 10 days, and underwent lumbar puncture as part of end-point assessment. Outcomes included clinical (mortality, rescue treatment) and parasitologic (clearance of trypanosomes) measures. At 18 months, success rates were 91.2% for the fexinidazole group and 97.6% for the NECT group, which was considered an acceptable margin by WHO. Furthermore, fexinidazole was well tolerated compared to NECT, and there were no treatment-related deaths. The number of deaths due to any reason was less than the 5.9% mortality rate reported with melarsoprol. The four serious adverse effects related to treatment included personality changes, psychosis, and hyponatremia, but the benefits of oral fexinidazole therapy outweighed these risks overall [55].

#### Conclusions

HAT is a fatal neglected tropical disease affecting mostly rural and remote parts of sub-Saharan Africa. Conventional treatments used for HAT are highly toxic and potentially fatal. The arsenic compound melarsoprol was the mainstay of therapy for late stage HAT since 1949 and is still the only treatment for *T. b. rhodesiense*. Newer drug protocols, such as NECT, were introduced more recently and carry a better safety profile than melarsoprol but still require hospitalization, sterile equipment, and trained health professionals. The rediscovery of oral fexinidazole and the recent clinical trial carried out for its utilization in both early and late stages of disease have been groundbreaking for HAT and will likely alter the landscape of the disease. Control strategies for HAT are moving away from traveling mobile teams in rural areas and shifting towards passive case detection at primary health centers. Moreover, molecular target testing, anti-tsetse vaccine research and other vector control programs are underway. Although there is still much work to be done in this area, it is possible that the advent of new molecular techniques and fexinidazole will aid WHO’s target of elimination by 2020.

## Hot topic 4: tafenoquine for *Plasmodium vivax* malaria

### Scope of impact: global

#### Background

Malaria is a life-threatening disease, transmitted through the bite of an infected female *Anopheles* mosquito. It is the most important human parasitic disease infecting approximately 200 million people worldwide and causing nearly 400,000 deaths each year [60]. The malaria parasites belong to the genus *Plasmodium*, and while there are over 100 recognized species of *Plasmodium*, only 6 have been recognized to infect humans including *P. falciparum, P. vivax, P. ovale, P. malariae,* and the two simian malarias, *P. knowlesi* and *P. simium* [60, 61]. Of these species *P. vivax* accounts for 20% of cases worldwide and causes almost half of all the cases of malaria outside of Africa [60, 62], primarily in Asia and Latin America [61]. Despite having low blood-stage parasitemia in comparison to *P. falciparum* malaria*, P. vivax* malaria is not benign. It has been shown to relapse within weeks to months following the primary infection, owing to formation of latent hypnozoites in the liver, which makes it difficult to eliminate and increases the risk of severe infection and fatal outcomes [63]. *P. vivax* malaria is an important infection of returning travelers associated with significant morbidity and mortality, yet new effective drugs for prevention, treatment and control have been inadequate and deeply neglected [64].

##### Presumptive anti-relapse therapy (PART) and radical cure

For decades the majority of therapeutics for malaria targeted *P. falciprium* with generalized applications including coverage of *P. vivax*. That said, several studies from across Asia and South America have shown that patients with *P. vivax* malaria treated with only chloroquine had a high risk of relapse. Data generated in the 1950’s in Korean war veterans with *P. vivax* demonstrated much lower relapse rates when the blood schizonticide (chloroquine or quinine) was overlapped with primaquine, and this spawned the use of primaquine as part of “radical cure”, which targets both replicating asexual blood-stages, and hypnozoites in the liver. Primaquine can also be used as a component of “presumptive anti-relapse therapy” (PART) in travelers to highly endemic areas for *P. vivax* for a prolonged period of time [65]. In this strategy, 14-days of primaquine is overlapped with the last 2 weeks of chemoprophylaxis (e.g., mefloquine or doxycycline). Post-travel PART is rarely prescribed in the average asymptomatic returned traveler to *P. vivax* risk areas due to concerns about its safety, efficacy, and inconvenience due to the need for glucose-6-phosphate dehydrogenase (G6PD) testing [63]. Although a two-week course of primaquine is burdensome and can cause potentially life-threatening hemolytic anemia in patients with G6PD deficiency, primaquine has been used effectively for relapse prevention of *P. vivax* since 1952 [63].

#### What changed in 2018?

##### Evolution of the 8-aminoquinoline hypnozoiticides

In 2018 the US FDA approved the use of a new hepatic schizonticidal and hypnozoiticidal 8-aminoquinoline called tafenoquine to prevent all malarias and prevent relapse from *P. vivax* and *P. ovale* [64]. Tafenoquine overcomes several of the limitations of primaquine in that it has approval for use as a chemoprophylactic, requires weekly dosing rather than daily, and has blood schizonticidal activity which minimizes the chances of breakthrough malarial infections [64]. Most of the advantages of tafenoquine over primaquine are conferred due to its relatively long half-life: 14 days versus 6 h for primaquine. Tafenoquine is being sold under the brand names Arakoda™ (60 Degrees Pharmaceuticals) and Krintafel™ (GSK) [64]. A comparison of the major chemoprophylactic agents is provided in Table 3.

**Table 3:** Comparison of malaria chemoprophylaxis options

Agent	Mefloquine	Doxycycline	Atovoquone-proquanil	Primaquine	Tafenoquine
Dosing	Weekly	Daily	Daily	Daily	Weekly
Radical cure	No	No	No	Yes	Yes
*P. falciparum* active	Yes	Yes	Yes	Yes	Yes
Pregnancy	Yes	No	No	No	No
G6PD safety	Yes	Yes	Yes	No	No
Children	Yes	No	Yes	Yes	?
Resistance	Yes	Yes	Yes	No	Unlikely
CYP dependent	No	No	No	Yes	?

Several randomized controlled trials have evaluated the safety and efficacy of tafenoquine as radical cure and found it to be non-inferior to primaquine [64–66]. The study participants in all studies were non-pregnant adults with G6PD activity levels > 70%. All arms of these studies included 3 days of chloroquine treatment, with tafenoquine (TQ) being administered as a single dose of 300 mg compared to primaquine (PQ) as 15-mg doses daily for 14 days (Table 4).

**Table 4:** Tafenoquine for radical cure of acute vivax malaria [64, 66, 80]

Trial type	Sample size	Regions	Placebo	% Recurrence free
RCT [64]	161	Asia, Africa, Americas	Yes	TQ = 89% PQ = 77% Placebo = 54%
RCT [66]	522	Asia, Africa, Americas	Yes	TQ = 62% PQ = 70% Placebo = 28%
RCT [80]	251	Asia, Africa, Americas	No	TQ = 73% PQ = 75%

*Abbreviations*: *PQ* Primaquine, *TQ* Tafenoquine

In addition, studies evaluating the protective efficacy of tafenoquine as a malaria chemoprophylactic agent are summarized in Table 5.

**Table 5:** Tafenoquine for malaria chemoprophylaxis [81–84]

Trial type / duration	Sample size	Regions / species	Placebo	Efficacy
RCT/6 months [81]	615 soldiers	Timor / Australia *P. falciparum* and *P. vivax*	No	N/A – 4 cases in TQ arm
RCT/15 weeks [82]	123 Adults	Kenya *P. falciparum*	Yes	86%
RCT/12 weeks [83]	231 Adults	Ghana *P. falciparum*	Yes	87% (equivalent to mefloquine)
Experimental exposure to *P. falciparum* [84].	16 Adults (4 controls)	Australia *P. falciparum*	Yes	100%

##### Future directions

Currently, tafenoquine is only recommended for those 16 years of age or older, and studies are currently underway to assess its safety and proper formulation for use in children (Nov 2019 - NCT02563496). The mechanism of tafenoquine metabolism in humans and whether it requires cytochrome P-450 or any of its isotypes, or other monoamine oxidases, is currently unknown. Additional research is required to answer this important mechanistic question, which will be essential for predicting drug interactions. Most of the safety and efficacy studies presented in the tables above have documented side-effects beyond 6 months of use, thus, future clinical trials will need to evaluate the long-term consequences of tafenoquine use (Oct 2020 – NCT03320174).

#### Conclusions

Tafenoquine is a newly approved long-acting 8-aminoquinoline with broad anti-malarial activity. A single dose has been shown to be non-inferior to 14-days of primaquine for a radical cure of *P. vivax* when administered in combination with chloroquine. However, like primaquine, tafenoquine demonstrates potential hemolytic toxicity in patients with G6PD deficiency, thus its use requires such quantitative testing in advance of use. It is also contraindicated as prophylaxis in pregnant and lactating women and potentially individuals with psychotic disorders. Tafenoquine is particularly useful for prophylaxis in travelers to areas with where *P. falciparum* and *P. vivax* are co-endemic.

## Hot topic 5: monkeypox outbreak in Nigeria

### Scope of impact: predominantly regional (West Africa, Europe), potentially global

#### Background

Monkeypox is caused by the monkeypox virus (MPXV), a member of the genus Orthopoxvirus within the Poxviridae family [67]. Other members of this genus that cause infection in humans include smallpox and cowpox [68]. MPXV was first discovered in monkeys in 1958 after an outbreak occurred at a monkey facility in Copenhagen, Denmark [69]. Soon after, it was discovered that MPXV had the proclivity to infect a variety of other mammals, including humans [70]. The first human case of monkeypox infection occurred in 1970 in the Democratic Republic of Congo (previously known as Zaire) [69].

##### Clinical presentation of monkeypox

Monkeypox presents similarly to the viral disease, smallpox, but tends to be less severe [71]. The initial presenting symptoms include fever, headache, fatigue, myalgia, and other non-specific prodromal symptoms [72]. A distinguishing feature is lymphadenopathy, which helps differentiate the disease from smallpox [67]. After 1–3 days of prodrome, a macular or vesicular rash may appear, often on the palms of hands or the soles of feet [73]. Typical incubation period is 1–2 weeks, while the illness lasts anywhere from 2 to 4 weeks [72]. Limited clinical data and anecdotal experience suggests that the Central African strain of MPXV may cause more severe disease than the West African strain of the disease.

##### Geographic distribution

In 1970, MPXV was first identified in a 9-year old boy in the DRC, a region where smallpox was thought to be eradicated from in 1968 [67]. Most of the cases since then have been reported in rural regions approximating rainforests. Thus, MPXV is considered endemic to regions of the Congo Basin and western Africa, particularly DRC ([67]; see https://www.who.int/news-room/fact-sheets/detail/monkeypox for map distribution of disease).

##### Transmission

MPXV can be transmitted through direct contact with the fluid from active lesions and through respiratory droplets [73]. It is generally transmitted from animal to human, thus constituting a zoonosis of particular importance to West Africa, however, human-to-human transmission has been documented as well. In Africa, human infections have been documented through handling of animals including monkeys, Gambian giant rats, and squirrels [67]. The route of zoonotic transmission is direct contact with blood, bodily fluids, or the cutaneous or mucosal lesions of an infected animal, or via ingestion of inadequately cooked meat of infected animals [67]. While unconfirmed, the reservoir for transmission is suspected to be the rodent population (e.g. rope squirrel and dormice) [73]. The natural route of animal acquisition is unknown, but likely reflects species specificity [70].

##### Treatment and prevention

Currently, there is no known treatment that is considered safe or effective, however, in retrospective studies, previous smallpox vaccination has demonstrated protective efficacy against the development of monkeypox [72]. While there has been no formal study on post-monkeypox exposure administration of smallpox vaccine, it has been used in outbreak situations and is felt to offer some protection. Antiviral treatments such as cidofovir have been used in vitro, but never evaluated in a clinical trial.

#### Landscape of monkeypox prior to 2018

##### US outbreak 2003

In 2003, monkeypox was introduced in the US following import of an exotic rodent from Ghana to Texas. Infected mice and squirrels were then distributed to an animal vendor facility in Illinois, at which some of the infected rodents’ cages were adjacent to the cages of prairie dogs destined for distribution across the US, an animal-to-animal transmission event that was ultimately suspected to be the cause of zoonotic transmission that year [74]. In total, 37 confirmed cases and 10 suspected cases of monkeypox occurred across 6 states following this sequence of importation and interspecific mixing events [74]. All human cases were linked to contact with prairie dogs, in the end, including cases where virus recovered from infected patients and that of the pet prairie dogs were confirmed to be MPXV [75]. As part of the outbreak control strategy, smallpox vaccine was offered to minimize transmission. Since 2003, there have been multiple small outbreaks outside DRC ranging in size from 1 to 20 cases.

#### What changed in 2018?

##### Nigerian outbreak 2017/18

The first cases in Nigeria occurred in September 2017 in the state of Bayelsa. At that time, there were 33 suspected cases in 7 states with a majority of the cases occurring in persons over the age of 20 [75]. From that point forth, the disease widely spread throughout the country. In 2018, transmissions escalated, leading to 45 confirmed cases and 114 suspected cases of monkeypox. There was one fatal outcome in an individual with advanced, untreated HIV. The Nigeria Centre for Disease Control (NCDC) coordinated monkeypox control activities such as regional monkeypox training, wildlife training, and enhanced surveillance in the states affected by the disease [76, 77]. NCDC ensured that suspected and confirmed cases were managed in designated health facilities adhering to National guidelines, including use of personal protective equipment and enhanced infection prevention and control strategies [76, 77].

##### Imported cases – UK and Israel

In the UK, there were 3 cases reported with the first 2 cases occurring in the month of September.2018. The first case was a Nigerian naval officer from Abuja visiting Cornwall for a training exercise [68]. After travelling from London to Cornwall, the officer developed a fever the next day and was sent to Public Health England for evaluation of lymphadenopathy and a rash near the groin [68]. Initially misdiagnosed as staphylococcal infection, the patient was treated with antibiotics, however, within days, the rash had spread to his torso, face, and arms. Re-evaluation prompted diagnostic testing of lesion fluid, and MPXV DNA was detected [78]. The second case was an Englishman who returned to London from a 22-day vacation to Southern Nigeria with fever, lymphadenopathy, and a maculopapular rash [68]. In September 2018, after the second case presented in the UK, an Israeli man who lived and worked in Port Harcourt became sick a week after his return from Nigeria, presenting with fever, vesicular rash, and lymphadenopathy [71]. The fourth exported case was a physician who cared for the second UK case in the hospital, and was identified in early October 2018. Again, monkeypox was not initially suspected and therefore full personal protection equipment was not worn to prevent infection [68]. Upon recognition of the third case, smallpox vaccination was administered for prevention of further transmission.

#### Conclusions

In summary, monkeypox is an orthopoxvirus similar to smallpox. It is mainly zoonotic, with rodents suspected as the principal reservoirs, and is transmitted through contact/respiratory droplets. The smallpox vaccine has been implemented as a prevention strategy due to theoretical and objective cross-protection benefits, much in the same way that cowpox was found to confer protection against smallpox in the days of Edward Jenner. Use of existing smallpox vaccine repositories is tenable for small, regional outbreaks of limited duration. However, with eradication of smallpox and comparatively small global vaccine stocks, reliance on a smallpox-based vaccine control strategy in the context of a larger more sustained or global outbreak would be extremely challenging. Similarly, new smallpox vaccine development is hindered by the lack of appropriate models for testing efficacy; regulatory testing requirements and restrictions; and the intermittency of market needs [79]. Thus, in light of recent outbreaks and exportation of monkeypox, specific vaccine development for this indication should be prioritized. Moreover, exportation of disease serves to remind clinicians that monkeypox remains part of the differential diagnosis in individuals presenting with widespread vesiculopustular rash, fever, and lymphadenopathy, particularly with a history of travel to West or Central Africa, understanding that with the globalization of the exotic animal trade, local transmissions could essentially occur anywhere.

#### 2018 in review: summary

To recap, 2018 was an eventful year in the field of tropical medicine, with significant developments in the areas of novel drug treatments (tafenoquine, fexinidazole) and novel licensures of existing drugs (ivermectin); deployment of novel vaccine strategies to influence outbreak control of vector-borne diseases (fractional YF Vax dosing); and travel-related globalization of zoonotic infectious diseases that are theoretically (monkeypox) or proven to be (YF) vaccine preventable. These new developments - both successes and challenges - should motivate continued attention, resourcing, and, ideally, innovation, in the control of zoonotic, vector-borne, and neglected tropical diseases. Although the pipeline to new drugs and vaccines for such indications is prolonged and expensive, 2018 has underscored how renewed focus on drugs already in development (tafenoquine); expansion of markets for orphan drugs licensed elsewhere (ivermectin); drugs that have been rediscovered (fexinidazole); novel methods to stretch a licensed but constrained resource (YF Vax); and laterality of vaccine deployment (smallpox vaccine) can disrupt traditional approaches to tropical infectious diseases and thereby advance the field.
